# Long‐Term Impact of Physical Activity on Mortality in Adults With Multimorbidity: A 12‐Year Cohort Longitudinal Study From the Survey on Health, Ageing and Retirement in Europe

**DOI:** 10.1002/jcsm.13695

**Published:** 2025-02-05

**Authors:** Nicola Veronese, Francesco Saverio Ragusa, André Hajek, Brendon Stubbs, Lee Smith, Mario Barbagallo, Ligia Juliana Dominguez, Luigi Fontana, Pinar Soysal, Shaun Sabico, Nasser M. Al‐Daghri

**Affiliations:** ^1^ Geriatric Unit, Department of Internal Medicine and Geriatrics University of Palermo Palermo Italy; ^2^ Biochemistry Department, College of Science King Saud University Riyadh Saudi Arabia; ^3^ Department of Health Economics and Health Services Research University Medical Center Hamburg‐Eppendorf, Hamburg Center for Health Economics Hamburg Germany; ^4^ Psychological Medicine, Institute of Psychiatry, Psychology and Neuroscience King's College London London UK; ^5^ Centre for Sport Science and University Sports University of Vienna Vienna Austria; ^6^ Centre for Health Performance and Wellbeing Anglia Ruskin University Cambridge UK; ^7^ Department of Medicine and Surgery Kore University of Enna Enna Italy; ^8^ Charles Perkins Center, Faculty of Medicine and Health University of Sydney Sydney New South Wales Australia; ^9^ Department of Endocrinology Royal Prince Alfred Hospital Sydney New South Wales Australia; ^10^ Department of Geriatric Medicine, Faculty of Medicine Bezmialem Vakif University Istanbul Turkey

**Keywords:** ageing, death, healthy ageing, longevity, mortality, multimorbidity, physical activity, SHARE study

## Abstract

**Background:**

While physical activity (PA) is known to reduce mortality in the general population, this relationship in individuals with multimorbidity (≥ 2 chronic conditions) is unclear. This longitudinal study aimed to investigate whether there is a long‐term association between PA levels and mortality rates over a 12‐year period in adults with multimorbidity.

**Methods:**

Data were obtained from eight waves of the Survey of Health, Ageing and Retirement in Europe (SHARE), from 28 European countries. PA levels were self‐reported via computer‐assisted personal interviews. Mortality during the follow‐up period was assessed using data obtained from caregivers through end‐of‐life interview. Multimorbidity was identified based on the presence of two or more 15 self‐reported chronic diseases/conditions. Cox's regression analysis, adjusted for potential confounders, was used to assess the association between PA level and mortality. *p*‐values were calculated using the Jonckheere–Terpstra test for continuous variables and the Mantel–Haenszel Chi‐square test for categorical variables, stratified by PA level.

**Results:**

The study included 9216 participants with multimorbidity (mean age 69 ± 10.1 years; 58.7% were women). Among those with multimorbidity, individuals with high PA level were significantly younger, more frequently men, less impaired in activities of daily living, less educated and less frequently obese than those with very low level of PA (*p* < 0.0001 for all comparisons). Over the 12 years of follow‐up, mortality incidence was three times higher in individuals with multimorbidity and very low PA levels than those with multimorbidity and high levels of PA. After adjusting for confounders, the risk of mortality was significantly lower for participants with moderately low PA levels (HR = 0.64; 95% CI: 0.59–0.71; *p* < 0.0001), moderately high PA levels (HR = 0.53; 95% CI: 0.47–0.60; *p* < 0.0001) and high PA levels (HR = 0.49; 95% CI: 0.43–0.55; *p* < 0.0001) compared to those with very low PA levels.

**Conclusions:**

Findings from the present study suggest that people with multimorbidity who had lower levels of PA were three times more likely to die prematurely after 12 years than adults with multimorbidity and higher levels of PA at baseline. These findings underscore the importance of promoting physical activity in adults with multimorbidity to reduce the risk of premature mortality. Future longitudinal research is required to confirm/refute our findings. Further, intervention studies are needed to understand whether increasing levels of physical activity in this population subsequently reduces mortality risk.

## 1 Background

As the global population ages, prioritizing the health and well‐being of older adults becomes increasingly crucial [1]. Among the many factors influencing quality of life and longevity in this demographic scenario, physical activity (PA) stands out as a key modifiable factor.

For people with multiple chronic conditions, taking part in various forms of PA plays a key role in enhancing health outcomes and lowering the risk of death. Recent research highlights that both moderate and vigorous physical activities are effective in managing such conditions. These activities not only boost physical function and mental well‐being but also improve overall quality of life for individuals dealing with several chronic diseases [2]. An Italian's prospective cohort study of 502 017 individuals showed how vigorous PA is a potentially neuroprotective lifestyle intervention for the prevention of Parkinson Disease [3]. Exercise (one domain of PA) programs combining strength training and aerobic workouts are particularly useful for older adults with conditions like sarcopenia, common in those with multiple chronic conditions.

Multimorbidity, defined as the presence of two or more chronic medical conditions [4], poses a significant and growing global challenge, impacting individuals, caregivers and society at large [4]. Those with multimorbidity face higher risks of premature mortality, frequent hospital admissions and prolonged hospital stays compared to individuals without multimorbidity [5].

Multimorbidity tends to develop 10 years earlier in communities with low socioeconomic status when compared to those with high socioeconomic status and is linked to early death, reduced physical ability, lower quality of life and higher use of healthcare services [6]. In recent years, numerous studies have been carried out to identify the clinical patterns of chronic conditions: These studies highlighted that depression, hypertension and diabetes are the most frequently co‐occurring chronic diseases, followed by mental health disorders and musculoskeletal conditions [7]. In addition to improving health system factors to support multimorbidity management, evidence suggests that healthcare providers directly involved in patient care should focus on the core principles of patient‐centred medicine and the chronic care model [8]. These approaches are essential for enhancing care for patients with multimorbidity.

Multimorbidity is often associated with a sedentary lifestyle and low levels of PA. For example, a European study of over 34 000 adults aged 50 and above from the Study on Global Ageing and Adult Health found that those with physical multimorbidity in middle‐income countries were much more likely to be sedentary than individuals without multimorbidity [9]. Conversely, maintaining a high level of PA throughout life has good merits, and it can reduce the risk of chronic diseases like heart disease, diabetes and certain cancers, which are common in older populations [10]. This is backed by several studies, including a comprehensive review that shows how higher PA levels are linked to a lower risk and better survival rates for various cancers [11]. Additionally, regular PA improves mental well‐being, functional abilities and social engagement, all crucial for preserving independence and overall health in older adults. However, PA in patients with multimorbidity has certain drawbacks. These include the risk of injury, particularly for those with musculoskeletal conditions like arthritis or osteoporosis, where high‐intensity exercise can lead to falls or fractures [12]. Overexertion is also a concern, especially for individuals with heart disease or COPD, as it can worsen symptoms or cause fatigue. Tailored exercise programs are essential, but the lack of access to specialized guidance may reduce their effectiveness.

The role of PA in reducing mortality rates is well documented. For instance, a Spanish study of 46 191 participants demonstrated that even previously inactive primary care patients could lower their mortality risk by becoming more physically active, even if their PA levels remained below recommended guidelines [13]. However, gaps in single‐time PA measurements could introduce biases and undermine the validity of these findings. The specific relationship between higher PA levels and mortality among individuals with multimorbidity remains underexplored but is potentially significant. To the best of our knowledge, only one country‐specific study in Brazil has examined this association. This study included 1019 participants aged 60 years or older and found that among those with multimorbidity, 70% did not achieved sufficient PA levels, yet higher PA levels were still beneficial in reducing mortality risk [14]. However, this study utilized a cross‐sectional design and thus the direction of the association could not be confirmed. Even a Chinese work supported this evidence in other context [15], but they only represented a population strictly limited to one country and they did not categorize PA into different levels.

Given this background, our research utilized data from the Survey on Health, Ageing and Retirement in Europe (SHARE) to investigate the potential connection between PA and mortality in individuals with multimorbidity. The present study is based on data from all European countries, dividing PA into four levels: very low, moderately low, moderately high and high. Findings from this study go beyond a mere academic pursuit. For researchers, findings will demonstrate the longitudinal role of PA in people with multimorbidity and potentially highlight the need for more research to explore how PA interventions impact health outcomes in this group; for clinicians, findings may highlight the value of including PA advice in the treatment of patients with multimorbidity, potentially making it a crucial part of managing their care; for patients, increasing PA levels may greatly improve life expectancy, encouraging them to actively participate in their health management. This study holds clinical significance as it provides evidence supporting the promotion of physical activity as a key intervention to lower mortality risk in a rapidly ageing population with multimorbidity, presenting a practical approach to enhancing both longevity and quality of life.

## 2 Materials and Methods

### 2.1 Population

The present analyses utilized data from multiple waves of the SHARE study, a multidisciplinary and cross‐national panel database that collects microdata on health, socio‐economic status and social and family networks. SHARE is a longitudinal study encompassing a representative sample of populations from Europe and Israel (SHARE Project, http://www.share‐project.org/organisation/share‐eric.html).

In this longitudinal cohort study, we used data from the following waves: Wave 1 (baseline, 2004–2006), Wave 2 (2006–2007), Wave 3 (2008–2009), Wave 4 (2011–2012), Wave 5 (2013), Wave 6 (2015), Wave 7 (2017–2018) and Wave 8 (2019–2020). The dataset includes numerous countries including Austria, Germany, Sweden, the Netherlands, Spain, Italy, France, Denmark, Greece, Switzerland, Belgium, Israel, Czech Republic, Poland, Luxembourg, Hungary, Portugal, Slovenia, Estonia, Croatia, Lithuania, Bulgaria, Cyprus, Finland, Latvia, Malta, Romania and Slovakia. Use of different waves guarantees a follow‐up period of 12 years.

SHARE employs a multistage clustered sampling method, ensuring it is nationally representative and able to investigate diverse cross‐country contexts as a ‘natural laboratory’. This approach spans various scientific disciplines and temporal settings, transforming the challenges of population ageing into opportunities and providing policymakers with reliable data for evidence‐based decisions. Data collection in SHARE is conducted through computer‐assisted personal interviewing (CAPI) (https://share‐eric.eu/fileadmin/user_upload/Release_Guides/SHARE_release_guide_8‐0‐0.pdf), where interviewers perform face‐to‐face interviews using laptops with the CAPI instrument in the participants' native languages [16].

The sample size for this study was based on the expected mortality rates in populations with multimorbidity and the association with PA levels. To detect a hazard ratio (HR) of at least 0.65 between different PA levels and mortality, with a power of 80% and a significance level of 0.05, a minimum of 8000 participants was estimated. This was calculated using standard methods for survival analysis with Cox proportional hazards regression, as detailed in the literature [17]. Given the expected mortality rate of around 35% during the 12‐year follow‐up period, the final sample of 9216 participants ensured adequate power to detect meaningful associations.

The primary inclusion criterion for SHARE is that participants must be aged 50 or older at the time of the survey. Additionally, the study often includes the partners of eligible individuals, regardless of the partner's age, to gather comprehensive household data. Exclusion criteria for SHARE typically involve individuals who are institutionalized or unable to participate due to severe health conditions that prevent them from completing the survey.

Multimorbidity patterns were based on the 15 self‐reported chronic diseases/conditions assessed at all time points in the SHARE. The presence of 14 of these conditions (heart attack, hypertension, hypercholesterolaemia, stroke or cerebral vascular disease, diabetes or high blood sugar, chronic lung disease [COPD], cancer, stomach or duodenal ulcer, Parkinson's disease, cataracts, dementia, other affective or emotional disorders, rheumatoid arthritis and osteoarthritis) was assessed using the question: ‘Has a doctor ever told you that you had/Do you currently have any of the conditions on this card?’. Multimorbidity was defined as the presence of two or more conditions, according to the most widely used definition. No comorbidity indexes were used.

### 2.2 Standard Protocol Approvals, Registrations and Patient Consents

The SHARE study undergoes continuous ethical review. Initial waves (1 through 4) received approval from the Ethics Committee of the University of Mannheim. Subsequent waves, starting from Wave 4 onward, have been overseen by Ethics Council of the Max Planck Society. Additionally, the implementation of SHARE in each participating country was reviewed and approved by the respective ethics committees or institutional review boards, as required. These comprehensive reviews ensured that all aspects of the SHARE study, including subprojects, adhered to legal norms and international ethical standards (https://share‐eric.eu/fileadmin/user_upload/respect_code_socio_economic_research.pdf). Prior to data collection, written informed consent was obtained from all participants.

### 2.3 Exposure: Physical Activity Level

Self‐reported PA levels were assessed using the CAPI questionnaire: The interviewers conducted face‐to‐face interviews using a laptop on which the CAPI instrument was installed (https://share‐eric.eu/data/data‐documentation/questionnaires). Personal interviews are necessary for SHARE because they make the execution of physical tests possible. PA levels were assessed through two questions: (1) Question br015 (engagement in sport or activities that are vigorous) and (2) Question br016 (participation in activities requiring a low or moderate level of energy) during an ideal week. SHARE conducted the accelerometer project to measure the level of PA and sedentary behaviour of the elderly with a sensor to gather data that are comparable across countries. The SHARE accelerometer study was conducted in 10 countries to ensure geographic variation: two northern (Denmark, Sweden), two southern (Italy, Spain), three eastern (Czech Republic, Poland, Slovenia) and three central (Belgium, France, Germany) European countries. For each of the countries, the defined target was a net sample of 200 participants. Based on the SHARE manual (https://share‐eric.eu/fileadmin/user_upload/Methodology_Volumes/SHARE_Methodenband_WEB_Wave8_MFRB.pdf), we created a variable to quantify PA levels as follows: Activity level = (2*(4 − br015) + (4 − br016)) [16]. PA levels were then categorized into four groups: activity level (0–1 = very low), (2–3 = moderately low), (4–7 = moderately high), and (8–9 = high) [16]. The agreement between self‐reported physical activity level and accelerometer was overall good (kappa = 0.86).

### 2.4 Outcome: Mortality

Mortality during the follow‐up period was assessed using data obtained from caregivers through end‐of‐life interviews. From the second wave SHARE surveys include an ‘end‐of‐life’ section. If a participant from the first wave of SHARE passed away between the two waves, an ‘end‐of‐life’ interview was conducted. During this interview, relatives, friends or neighbours provided details about the deceased's final year. This module offers information on the date, cause and location of death. Mortality were tracked over a 12‐year follow‐up period across multiple waves of the SHARE study, from Wave 1 (2004–2006) to Wave 8 (2019–2020).

### 2.5 Covariates

To examine the association between PA level and mortality, several baseline confounding factors were considered. These factors, identified from the literature and available in the SHARE study [18], included age, sex, activities of daily living (ADL) limitations, body mass index (BMI), current smoking status, marital status, highest level of education obtained and country of residence.

Age was measured as a categorical variable (50–64, 65–74, and 75 and over), and sex was specified as a binary indicator (women vs. men). The ADL index measured limitations in daily activities, highlighting challenges in self‐care tasks such as dressing, walking, grooming, eating, getting in and out of bed and using the toilet, which are essential for maintaining independence. The modified ADL indices used in the SHARE questionnaire cover six activities (dressing, including putting on shoes and socks, walking across a room, bathing or showering, eating, such as cutting up your food, getting in or out of bed, using the toilet, including getting up or down). Participants reporting difficulties with these activities for more than 3 months were included. The index summed affirmative responses, resulting in scores from 0 to 6, where higher scores indicate fewer difficulties and better mobility and autonomy. Marital status was defined as married or in a partnership, not married or single or widowed. Smoking status was categorized as never smoked, ex‐smoker, or current smoker. Level of education was established based on the International Standard Classification of Education (ISCED), aggregated into three categories: (1) ISCED 0–1: no education or a low level of education, (2) ISCED 2–4: intermediate level of education, and (3) ISCED 5–6: higher level of education. BMI was calculated from self‐reported height and weight (weight in kg/m^2^) and categorized as underweight (< 18.5), normal weight (18.5 to 24.9), overweight (25 to 29.9) or obese (≥ 30).

### 2.6 Statistical Analysis

Means and standard deviations (SD) were used to describe continuous variables, while percentages were used for categorical variables. Levene's test was used to test the homoscedasticity of variances and to measure homogeneity, and if its assumption was violated, Welch's ANOVA was applied. *p*‐values were calculated using the Jonckheere–Terpstra test for continuous variables and the Mantel–Haenszel chi‐square test for categorical variables, stratified by PA level.

The association between PA level at Wave 1 in individuals with multimorbidity at baseline and mortality was analysed using a Cox's regression analysis, adjusting for the aforementioned confounding variables and changes in PA level across waves. Factors included in the multivariate analysis were initially based on previous literature [18]. Collinearity among factors was assessed to exclude covariates with a variance inflation factor (VIF) over two, but no factors were excluded for this reason. The results were reported as hazard ratios (HRs) with their 95% confidence intervals (CIs). To test the robustness of our results, we examined the interaction between PA level and mortality for several moderators, including age (median value), sex, number of comorbidities (categorized in 2, 3 or ≥ 4 conditions), but these factors did not modify our findings.

All statistical analyses were two‐tailed, and a *p*‐value < 0.05 was considered statistically significant using Bonferroni's correction. All analyses were performed using SPSS 26.0 version software.

## 3 Results

**FIGURE 1:**
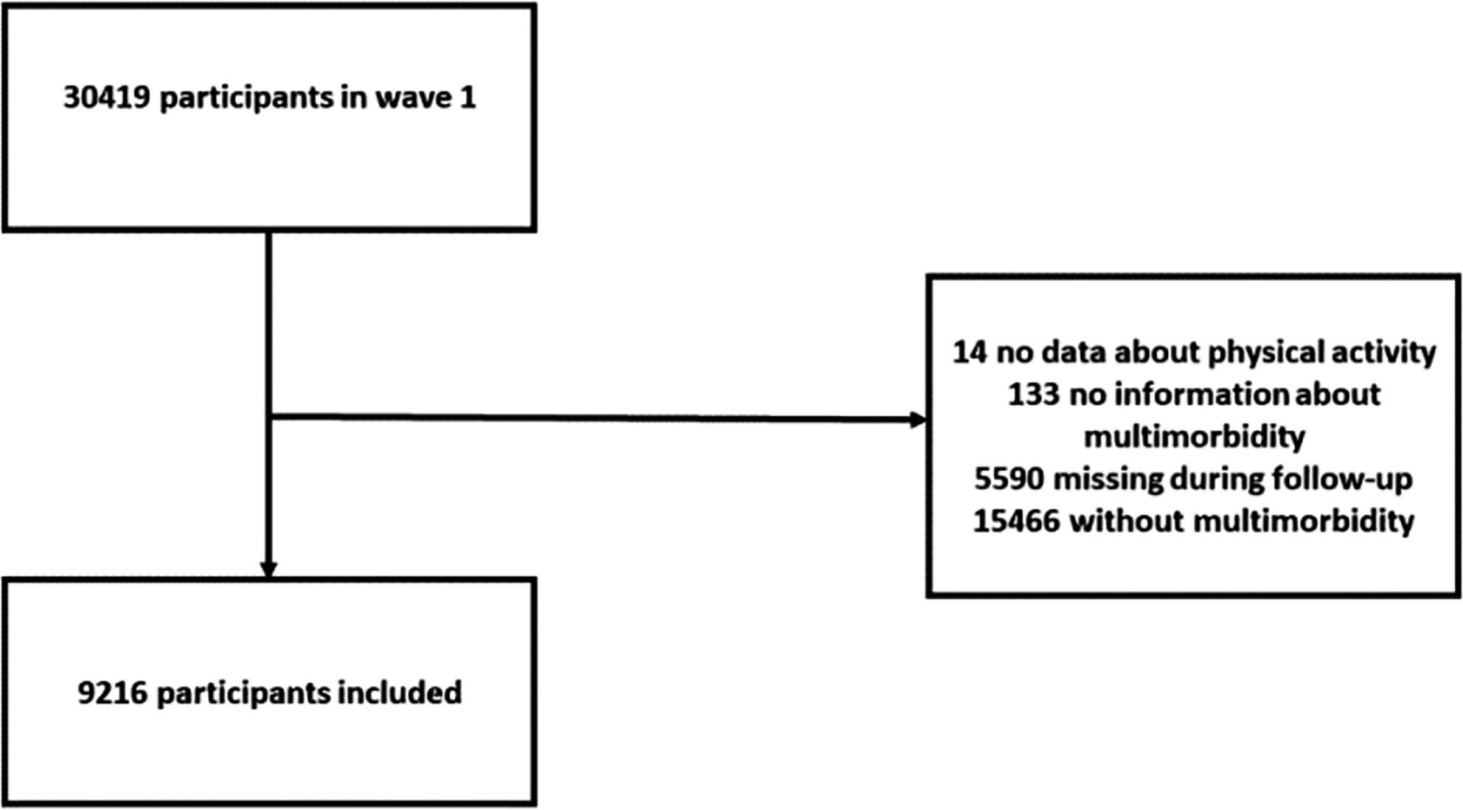
Flowchart of participants included in the study.

Figure 1 shows the research flow‐chart. Out of the 30 419 participants initially considered in wave 1 of the SHARE study, we excluded 14 due to missing PA data, 133 due to unavailable multimorbidity data and 5590 were lost during follow‐up. A total of 15 466 participants did not have multimorbidity, leaving 9216 participants for the present analyses. Participants had a mean age of 69 ± 10.1 years, and they were predominantly women (58.7%). Most had two comorbidities (*n* = 50.4%), while 26.8% had three, and 22.8% more than three.

Table 1 presents the participants' characteristics by PA level and includes the ICD classification of diseases. Participants with high PA levels were significantly younger, more likely to be men, less impaired in ADL, less educated and less frequently obese compared to those with very low PA levels (*p* < 0.0001 for all comparisons). Additionally, individuals with high PA levels reported higher alcohol consumption in the past 6 months, were more often smokers and were more frequently married (*p* < 0.0001 for all comparisons). Regarding medical conditions, participants with low PA levels had a higher prevalence of chronic conditions, including heart attack, stroke, diabetes, arthritis, osteoporosis, COPD, Parkinson's disease and hip or femoral fractures (*p* < 0.0001 for all, except asthma, *p* = 0.01). However, no significant differences were observed in the prevalence of hypertension, asthma, cancer or gastrointestinal ulcers (*p* > 0.05 for these comparisons).

**TABLE 1:** Sample characteristics by physical activity level.

Parameters	Very low	Moderately low	Moderately high	High	*p*
*N*	1876	3195	1817	2328	
Age (years)	73.5 ± 10.8	70 ± 9.8	66.9 ± 8.9	65.4 ± 9.0	< 0.0001
BMI (kg/m^2^)	26.3 ± 8.0	26.4 ± 6.3	26.8 ± 5.1	26.6 ± 5.2	< 0.0001
Women	1208 (64.4)	1929 (60.4)	1089 (59.9)	1185 (50.9)	< 0.0001
1 or more ADL limitations	832 (44.4)	524 (16.4)	160 (8.8)	147 (6.3)	< 0.0001
Actual smoke at the baseline	279 (14.9)	437 (13.7)	283 (15.6)	383 (16.5)	< 0.0001
Obesity (BMI > 30 kg/m^2^)	452 (24.1)	694 (21.7)	404 (22.2)	469 (20.2)	< 0.0001
Married	1039 (55.4)	2076 (65)	1276 (70.2)	1713 (73.6)	< 0.0001
First level of education	598 (31.9)	872 (27.3)	539 (29.7)	549 (23.6)	< 0.0001
Alcohol consumption in the last 6 months	745 (39.7)	2003 (62.7)	1230 (67.7)	1693 (72.7)	< 0.0001
Medical conditions ever reported (ICD classification)					
Heart attack (I21.9)	688 (36.7)	941 (29.5)	420 (23.1)	505 (21.7)	< 0.0001
Hypertension (I10)	1172 (62.5)	1959 (61.3)	1119 (61.6)	1436 (61.7)	0.87
High cholesterol (E78.00)	660 (35.2)	1375 (43)	869 (47.8)	1166 (50.1)	< 0.0001
Stroke (I63.9)	292 (15.6)	266 (8.3)	113 (6.2)	127 (5.5)	< 0.0001
Diabetes (E119)	514 (27.4)	699 (21.9)	385 (21.2)	488 (21)	< 0.0001
Chronic lung disease (J44.9)	281 (15)	346 (10.8)	169 (9.3)	200 (8.6)	< 0.0001
Asthma (J45909)	204 (10.9)	300 (9.4)	141 (7.8)	208 (8.9)	0.01
Arthritis (M13.80)	883 (47.1)	1321 (41.3)	628 (34.6)	727 (31.2)	< 0.0001
Osteoporosis (M81.0)	404 (21.5)	554 (17.3)	279 (15.4)	333 (14.3)	< 0.0001
Cancer (C80. 1)	200 (10.7)	332 (10.4)	164 (9)	241 (10.4)	0.34
Gastrointestinal ulcer (K25. 9)	242 (12.9)	390 (12.2)	235 (12.9)	286 (12.3)	0.81
Parkinson's disease (G20. A1)	432 (23)	614 (19.2)	258 (14.2)	338 (14.5)	< 0.0001
Hip or femoral fracture (M84. 459A/S72301A)	156 (8.3)	139 (4.4)	62 (3.4)	62 (2.7)	< 0.0001

*Note:* Data presented as *N* (%) except for age and BMI, presented as mean ± SD; significant at *p* < 0.05.

Abbreviations: ADL: activity daily living; BMI: body mass index.

**FIGURE 2:**
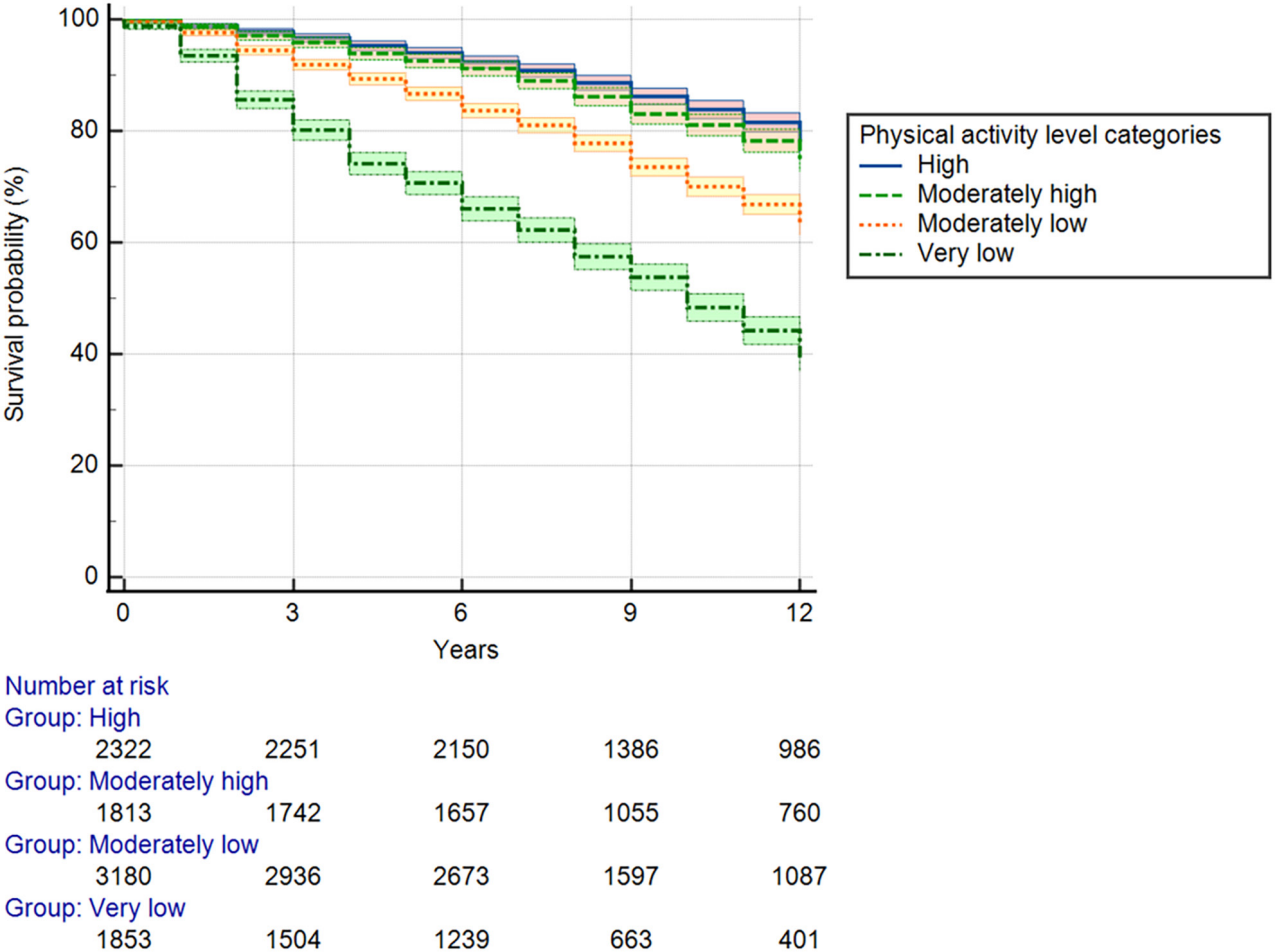
Survival curves by physical activity levels over 12 years.

During the follow‐up, 34.7% of the initial population died, with a global incidence rate of 3400 deaths per 100 000 persons‐year (Table 2 and Figure 2). The crude incidence rate in the very low PA group is three times that of the high PA group. After adjusting for covariates, including age, sex, ADL limitations, BMI, smoking status, marital status, education level and country of residence, participants with higher PA levels showed a significantly reduced mortality risk compared to those with very low PA. Specifically, the hazard ratios (HR) were as follows: moderately low PA (HR = 0.64; 95% CI: 0.59–0.71; *p* < 0.0001), moderately high PA (HR = 0.53; 95% CI: 0.47–0.60; *p* < 0.0001) and high PA (HR = 0.49; 95% CI: 0.43–0.55; *p* < 0.0001). These findings highlight the protective effect of increasing PA levels on mortality risk during the follow‐up period.

**TABLE 2:** Association between mortality and physical activity.

Labels	Incidence rate (per 100 000 persons‐years)	Crude model	*p*	Fully adjusted model^a^	*p*
Very low	6866	1 [reference]	< 0.001 (for trend)	1 [reference]	< 0.001 (for trend)
Moderately low	3529	0.49 (0.45–0.53)	< 0.001	0.64 (0.59–0.71)	< 0.001
Moderately high	2316	0.31 (0.28–0.35)	< 0.001	0.53 (0.47–0.60)	< 0.001
High	2006	0.27 (0.24–0.30)	< 0.001	0.49 (0.43–0.55)	< 0.001

*Note:* Data are reported as hazard ratios (HRs) with their 95% confidence intervals (CIs).

Abbreviations: ADL: activity daily living; BMI: body mass index.

^a^ The fully adjusted model was adjusted for age, sex, ADL limitations, BMI, current smoking status, marital status, highest level of education obtained, and country of residence.

Interactions between PA level and mortality risk were tested for median age (*p* for interaction = 0.38), sex (*p* for interaction = 0.21) and number of comorbidities (*p* for interaction = 0.98), all of which did not modify our findings.

## 4 Discussion

Our study represents one of the pioneering multinational efforts to investigate the longitudinal relationship between PA levels and mortality in individuals with multimorbidity. We found that higher levels of physical activity could significantly reduce mortality risk over an average 12‐year follow‐up period, even among those dealing with multiple chronic conditions.

Multimorbidity poses significant challenges for older adults, exacerbating symptoms, complicating treatment regimens, and increasing the risk of adverse drug reactions due to polypharmacy [19]. Consequently, individuals with multimorbidity often experience higher rates of hospitalizations, extended recovery times and elevated likelihood of needing long‐term care. According to a large systematic review, multimorbidity affects more than one in three individuals, highlighting its widespread prevalence and impact on health outcomes [20].

Increased PA has been found to be beneficial in improving function for older adults dealing with multimorbidity and frailty [21]. A pooled analysis of 507 surveys across 163 countries and territories highlighted that a third of adults worldwide do not meet recommended PA levels, and most countries are unlikely to achieve the global target set for 2030 [22].

An interesting finding was that people with higher PA levels reported greater alcohol consumption in the previous 6 months during the baseline evaluation. Conversely, a Brazilian study of 60 202 participant revealed that adults who consumed alcohol weekly were more likely to be classified as physically active compared to those who did not consume alcohol, while almost daily alcohol consumption was associated with lower PA [23]. Furthermore, individuals reporting higher PA levels were more frequently smokers than those with lower activity levels, possibly due to greater independence and reduced disability. A systematic review of 5733 older adults across six studies found a significant association between PA and increased independence [24].

In the present study, it is noteworthy that the PA level significantly decreased across waves. The percentage of people engaging in very low PA increased nearly three‐fold from the first to the last wave, indicating a potentially prevalent and exponentially rising sedentary behaviour trend. Conversely, participants with initially high activity levels showed a decrease in their activity from the first to the eighth wave. This decline could be attributed to the ageing process over the 12‐year follow‐up period, which typically diminishes physical fitness, including strength, endurance, agility and flexibility, thereby impacting daily activities and overall functionality among older adults [25].

Research supports our findings that higher PA levels may lower mortality rates. A British study of 14 599 participants showed that those with increasing PA trajectories over time had lower risks of all‐cause mortality compared to consistently inactive individuals, although the use a postal questionnaires for follow‐up may have impacted data quality [26]. Similarly, a Taiwanese study involving 481 688 individuals demonstrated that alternating between sitting and standing at work, coupled with an additional extra 15 to 30 min of daily PA, can mitigate the negative effects of prolonged occupational sitting, thereby reducing mortality risk [27]. However, modifications to their self‐reported physical activity questionnaire during extended follow‐up periods could potentially lead to measurement inconsistencies.

Our results show that, after adjusting for several potential confounding factors, individuals with multimorbidity who reported moderately low, moderately high and high levels of PA had a 36%, 47% and 51% reduced mortality risk, respectively, compared to those with low levels of PA. PA may reduce mortality through several mechanisms. First, engaging in regular PA boosts mitochondrial function, enhancing energy production and decreasing oxidative stress, thereby protecting cells from damage. PA also increases the expression of antioxidant enzymes, which help neutralize harmful free radicals [28]. Second, PA regulates inflammation by lowering pro‐inflammatory cytokine levels and raising anti‐inflammatory interleukins. It also stimulates autophagy, the process by which cells remove damaged proteins and organelles, ensuring cellular health [29]. Thirdly, PA enhances insulin sensitivity, aiding in blood glucose regulation and potentially slowing the accumulation of molecular damage [30]. Together, these hormonal and molecular changes reduce the risk of chronic diseases and decrease overall mortality.

Our research findings should be considered with several limitations in mind. First, the SHARE study predominantly includes Caucasian participants, which may exclude populations that experience higher levels of discrimination and social barriers to PA; collecting data across different countries or regions brings challenges in standardizing data collection methods, as cultural differences can affect how participants perceive and respond to surveys. Second, the self‐reported assessment of PA may suffer from some bias in several dimensions that are especially relevant in the SHARE sample: Old age and limitations in mobility make it even more difficult to compare the respondents' answers concerning their physical activities, because the interpretation of ‘moderate’ and ‘vigorous’ activities might differ due to individual health conditions and living situations. Third, we lacked information on the duration of the diseases considered in the multimorbidity variable. Fourth, other important aspects of healthy ageing, such as dietary habits, were not assessed. Lastly, our main exposure variable, PA level, was based on self‐reported data. Self‐reported data, especially on sensitive topics like health conditions or PA, can be inaccurate due to recall bias, misreporting or misunderstanding of the questions. It is well established that objective measures of PA, such as accelerometery, are more accurate: Research data have demonstrated that the use of accelerometers is feasible, provides reproducible findings and offers valid measures of PA [31]. Accelerometers accurately detect sedentary behaviour and capture information on the intensity, duration and frequency of PA [32].

In conclusion, this study underscores the crucial impact of PA in lowering mortality risk among individuals with multimorbidity over a 12‐year follow‐up. Even for those with multimorbidity, higher levels of PA were linked to significantly reduced mortality rates. Although PA levels naturally decrease with age, staying physically active contributes to better health outcomes and increased longevity. The study's limitations include the reliance on self‐reported data and a predominantly Caucasian sample, emphasizing the need for more diverse populations and objective data in future studies. Promoting customized PA programs remains vital for managing multimorbidity and supporting healthy ageing.

## Ethics Statement

The SHARE study is subject to continuous ethics review. During Waves 1–4, SHARE was reviewed and approved by the Ethics Committee of the University of Mannheim. Wave 4 and the continuation of the project were reviewed and approved by the Ethics Council of the Max Planck Society. In addition, the country implementations of SHARE were reviewed and approved by the respective ethics committees or institutional review boards whenever this was required. The numerous reviews covered all aspects of the SHARE study, including subprojects and confirmed the project to be compliant with the relevant legal norms and that the project and its procedures agree with international ethical standards (http://www.share‐project.org/fileadmin/pdf_documentation/SHARE_ethics_approvals.pdf).

## Consent

Written informed consent was obtained from all participants in the study before data were collected.

## Conflicts of Interest

B.S. is on the Editorial Board of *Ageing Research Reviews*, *Mental Health and Physical Activity*, the *Journal of Physical Activity and Health* and the *Brazilian Journal of Psychiatry*. Brendon has received honorarium from a co‐edited book on exercise and mental illness (Elsevier), associated training and unrelated advisory work from ASICS and FitXR LTD. The other authors declare no conflicts of interest.

## Data Availability

Data are available upon request to the corresponding author.
